# Continuous table movement for peripheral MRA with matrix coils at 3.0 T

**DOI:** 10.1186/1532-429X-11-S1-P282

**Published:** 2009-01-28

**Authors:** Harald Kramer, Peter Schmidt, Christian Glaser, Maximilian F Reiser, Karin A Herrmann

**Affiliations:** 1 University Hospital of Munich, Munich, Germany; 2 Siemens Medical Solutions, Erlangen, Germany

**Keywords:** Peripheral Arterial Occlusive Disease, Matrix Coil, Fontaines Stage, Contrast Agent Bolus, Reconstructed Voxel Size

## Introduction

To compensate for the restricted field of view (FoV) of standard MRA systems for larger anatomical areas such as the peripheral arteries of the lower body part, a multistep approach was introduced and successfully implemented for MRA of the lower limb. However, even concepts of a sequential multi-step MRA with a double CA bolus include several disadvantages. The planning and separate data acquisition of multiple steps for both non-enhanced and contrast enhanced imaging is time consuming. To face and overcome these limitations, a novel technique for continuous table movement and data acquisition for MRA has been introduced at 3.0 Tesla.

## Purpose

To compare a standard step-by-step and a newly developed continuous table movement (ct) technique for peripheral MRA at a 3.0 Tesla MR System equipped with a matrix coil system.

## Methods

**Figure 1:**
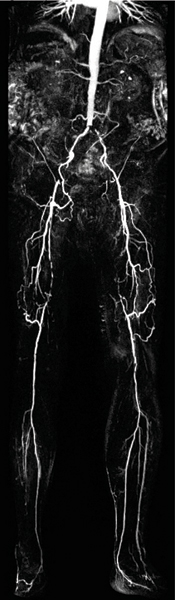
**CT-MRA dataset with excellent image quality without any venous enhancement or artefacts**. Clear depiction of several atherosclerotic changes like occlusion of the superficial femoral artery on both sides.

Peripheral MRA with continuous table movement acquires one large FoV with up to 130 cm in patient z-axis. Before acquiring the contrast enhanced dataset vessel scout images as well as a non contrast enhanced dataset for later subtraction are acquired. We included 14 consecutive patients referred for peripheral MRA with clinical symptoms of peripheral arterial occlusive disease (PAOD) Fontaines stage II – IV. All of them underwent both step-by-step MRA and ct-MRA in one session. Patients with impaired renal function (calculated GFR < 30 ml/min) were not included. All exams were performed on a 3.0 T MR System (Magnetom Verio). Maximal contrast agent (CA) volume was 31.5 ml (1.5 ml testbolus, 15 ml/MRA technique). For both techniques the same monophasic CA injection protocol was used, 15 ml of standard 0.5 molar CA were injected at a flow rate of 1 ml/s directly followed 25 ml of saline also at 1 ml/s. Spatial resolution of the ct-MRA datasets is technically limited to a reconstructed voxel size of 1.0 × 1.0 × 1.3 mm^3^ for the entire FoV. Acquired voxel sizes differ throughout the FoV due to a technique called "variable resolution" helping to increase resolution in the most distal part of the FoV. Step-by-step MRA reached a spatial resolution between 1.4 × 1.1 × 1.2 mm3 and 0.9 × 0.9 × 0.9 mm^3^ in the most distal calf station. First ct-MRA datasets were read and findings thereafter correlated with the step-by-step MRA datasets. See Figure 1.

## Results

All datasets could be evaluated. Due to the absence of multiple localizers and subtraction masks examination time was considerably shorter when using the ct-MRA technique. Relevant findings detected by step-by-step MRA were also detected by ct-MRA. Different results between step-by-step MRA and ct-MRA occurred in the differentiation between no vessel-wall changes/slight atherosclerotic changes and high grade stenosis/occlusion respectively.

## Conclusion

MRA with continuous table movement is an easy applicable technique for imaging peripheral vessels without the need for planning different steps and FOV positioning, thus examination time can be reduced considerably. However, the slightly reduced spatial resolution compared to standard step-by-step MRA is a drawback especially in the most distal calf vessels.

